# Acupuncture and moxibustion for chronic fatigue syndrome in traditional Chinese medicine: a systematic review and meta-analysis

**DOI:** 10.1186/s12906-017-1647-x

**Published:** 2017-03-23

**Authors:** Taiwu Wang, Cong Xu, Keli Pan, Hongyan Xiong

**Affiliations:** Department of Epidemiology, College of Preventive Medicine, Third Military Medical University, Gaotanyan Road 30, Shapingba District, Chongqing, 400038 China

**Keywords:** Acupuncture and moxibustion, Chronic fatigue syndrome, Traditional meta-analysis, Network meta-analysis, Acupoint

## Abstract

**Background:**

As the etiology of chronic fatigue syndrome (CFS) is unclear and the treatment is still a big issue. There exists a wide range of literature about acupuncture and moxibustion (AM) for CFS in traditional Chinese medicine (TCM). But there are certain doubts as well in the effectiveness of its treatment due to the lack of a comprehensive and evidence-based medical proof to dispel the misgivings. Current study evaluated systematically the effectiveness of acupuncture and moxibustion treatments on CFS, and clarified the difference among them and Chinese herbal medicine, western medicine and sham-acupuncture.

**Methods:**

We comprehensively reviewed literature including PubMed, EMBASE, Cochrane library, CBM (Chinese Biomedical Literature Database) and CNKI (China National Knowledge Infrastructure) up to May 2016, for RCT clinical research on CFS treated by acupuncture and moxibustion. Traditional direct meta-analysis was adopted to analyze the difference between AM and other treatments. Analysis was performed based on the treatment in experiment and control groups. Network meta-analysis was adopted to make comprehensive comparisons between any two kinds of treatments. The primary outcome was total effective rate, while relative risks (RR) and 95% confidence intervals (CI) were used as the final pooled statistics.

**Results:**

A total of 31 randomized controlled trials (RCTs) were enrolled in analyses. In traditional direct meta-analysis, we found that in comparison to Chinese herbal medicine, CbAM (combined acupuncture and moxibustion, which meant two or more types of acupuncture and moxibustion were adopted) had a higher total effective rate (RR (95% CI), 1.17 (1.09 ~ 1.25)). Compared with Chinese herbal medicine, western medicine and sham-acupuncture, SAM (single acupuncture or single moxibustion) had a higher total effective rate, with RR (95% CI) of 1.22 (1.14 ~ 1.30), 1.51 (1.31–1.74), 5.90 (3.64–9.56). In addition, compared with SAM, CbAM had a higher total effective rate (RR (95% CI), 1.23 (1.12 ~ 1.36)). In network meta-analyses, similar results were recorded. Subsequently, we ranked all treatments from high to low effective rate and the order was CbAM, SAM, Chinese herbal medicine, western medicine and sham-acupuncture.

**Conclusions:**

In the treatment of CFS, CbAM and SAM may have better effect than other treatments. However, the included trials have relatively poor quality, hence high quality studies are needed to confirm our finding.

**Electronic supplementary material:**

The online version of this article (doi:10.1186/s12906-017-1647-x) contains supplementary material, which is available to authorized users.

## Background

CFS is a wide-spread agnogenic disease [1] characterized by unexplained fatigue that lasts for at least six months and accompanied by four or more of the following symptoms: unrefreshing sleep, lengthy malaise after exertion, impairment of concentration or short-term memory, sore throat, tender lymph nodes, multi-joint and muscle pain and headaches [2]. CFS affects work and life seriously, at the same time causes heavy societal burden with an incidence of 0.2% ~ 2.2% in adults [3, 4], 0.1% ~ 0.5% in adolescents [5] in western countries and 1.9% in Beijing and 3% in Hong Kong, China [6]. American CDC reported a higher prevalence, with 2.3% in children and 2.3% in adolescents (http://www.cdc.gov/cfs/pediatric/index.html). Though many theories are used to explain CFS, the aetiology is still unclear and no major progress has been made in therapy. So far, in treatment of CFS, cognitive behavior [7–9] and graded exercise therapies [10, 11] are thought to be effective in relief of symptoms, and the curative effect of western medicines isn’t promising. For instance, the effects of anxiolytics, corticosteroids, dietary supplements, evening primrose oil, homeopathy, magnesium (intramuscular), nicotinamide adenine dinucleotide (oral) and prolonged rest are unclear [12]. Suggestions on treatment have been made in a number of countries [13, 14], but their effect still needs to be further studied [15].

CFS has no clear mechanism, with its complex symptoms. As the treatment effectiveness by western medicine were limited overall, some researchers also begun to shift their focus towards complementary and alternative medicine. Acupuncture and moxibustion in traditional Chinese medicine (TCM) [16] comes to the attention of researchers. Acupuncture means the insertion of needles into different parts or points of the body and moxibustion mans the use of burning moxa to stimulate certain parts or points of the body, while moxa is usually made from a special herb named argy wormwood leaf. As the development of acupuncture and moxibustion, acupuncture and moxibustion have many different forms, for example, both auricular acupuncture and electroacupuncture belong to acupuncture. In the realm of traditional Chinese medicine, the human body is a whole organism. Though the mechanism of CFS treated by acupuncture and moxibustion is not fully explained, the treatment is effective in relief of symptoms [17, 18]. The acupuncture and moxibustion treatment can adjust organ function of traditional Chinese medicine, such as the zhang and fu, and have various ways in which they can be chosen depending on the characters of patients.

Even though some systematic review and meta-analysis were done before [19–21], there were also some limitations. The Alraek’s study [21] didn’t include acupuncture or moxibustion, therefore its relevance to this review is unclear, and the other two studies [19, 20] didn’t conduct meta-analysis for acupuncture and moxibustion. Therefore comprehensive meta-analysis evidence is still not available that showed the therapeutic effect of comparing acupuncture and moxibustion with many others treatments. The network meta-analysis combines the comparisons of treatments not only directly addressed within any of the individual trials, but also incorporated the indirect comparisons constructed from two trials that share one treatment in two studies. This kind of meta-analysis makes full use of the within-trial randomized treatment comparison of each trial, while combining all available comparisons between treatments. This study attempts to evaluate the effect of acupuncture and moxibustion on CFS systematically compared with other treatments by traditional and network meta-analysis.

## Methods

### Search strategy and study selection

We searched through the databases of PubMed, EMBASE, Cochrane library, Chinese Biomedical Literature Database (CBM), China National Knowledge Infrastructure (CNKI), China Master Theses Full-text Database (CMTD), China Doctor/Maseter’s Dissertations (CDMD) Full-text Database. The last search for all databases was updated to May 2016. We used the combined method of MeSH Term and free words by applying the following terms: acupoint injection, acupuncture, acupuncture and moxibustion, acupuncture points, acupuncture therapy, auricular acupuncture, auricular plaster, body acupuncture, coiling dragon needling, dermal needle, dry needling, ear acupuncture, ear seed pressure, Electro-acupuncture, embedding, embedding therapy, fire needle, moxibustion, *panlongci*, percussopuncture, point injection, pricking blood, scalp acupuncture, trigger points, meridians and chronic fatigue syndrome, chronic fatigue, fatigue syndrome, Myalgic Encephalopathy in PubMed, EMBASE, Cochrane library and relevant Chinese words in CBM and CNKI.

### Inclusion and exclusion criteria

#### Inclusion criteria

(1) Randomized controlled trials (RCTs) in English or Chinese were included regardless of whether published or unpublished. (2) The patients were diagnosed under clear criteria (CDC 1988 [22] or CDC 1994 [2]); (3) RCT interventions adhered to the following treatment strategies: the experimental group received combined acupuncture plus moxibustion or acupuncture or moxibustion alone, while the control group received treatment with Chinese herbal medicine, western medicine, placebo treatment, acupuncture or moxibustion alone (for combined acupuncture plus moxibustion). (3) Clinical efficacy (cured, markedly effective, and effective) and invalid evaluation were used as the end-point [23], and the criteria for invalid evaluation was the syndrome score was reduced by less than 30% or 1/3 [23].

#### Exclusion criteria

The exclusion criteria included the following: (1) non-RCTs or duplicate publications; (2) animal studies; (3) case reports and reviews; (4) clinical research studies that compared different kinds of acupuncture or moxibustion; (5) the treatment was combined with others treatments than acupuncture and moxibustion; (6) the study with western medicine which were clearly not recommended according to the latest NICE guideline, such as glucocorticoids, mineralocorticoids (https://www.nice.org.uk/guidance/cg53/chapter/1-Guidance#genera-management-strategies-after-diagnosis); (7) the criteria of invalid evaluation was not based on syndrome score reduction by less than 30% or 1/3.

### Quality assessment

The quality assessment of all studies included in this review was independently evaluated by two reviewers (Wang TW and Xu C) using the Cochrane Collaborations tool [24]. Seven criteria were applied: (1) random sequence generation, (2) allocation concealment, (3) blinding of participants and personnel, (4) blinding of outcome assessment, (5) incomplete outcome data, (6) selective reporting and (7) other bias (defined as baseline data comparability). For each item, the evaluation was denoted as low, high or unclear risk according to the descriptions of the method in each study. Any disagreement was resolved by discussion with the third author (Xiong HY).

### Data extraction and analysis

Related data such as title, first author, year of publishing, study design, intervention of each group, quality of the study, diagnosis criteria, number of participants, range of participants’ age, outcomes, treatment duration and adverse events were independently extracted by two reviewers (Wang TW and Xu C) using inclusion criteria. Disagreements were resolved by discussion between the two reviewers and by seeking the opinion of the third author (Xiong HY) if necessary.

Traditional meta-analysis was adopted for direct comparison. Random effects model was adopted for overall and subgroup analysis if obvious heterogeneity existed, otherwise fixed effects model. Furthermore, both models were adopted to test the difference of the two models for sensitivity analysis. At the same time, sensitivity analysis was finished by removing any single trial in each group. Statistical heterogeneity was evaluated by the Cochran’s Chi-squared test (with *P* < 0.10 indicating statistically significant heterogeneity) and the statistic *I* ^2^ [25] (The heterogeneity might not be important with *I* ^2^ of 0 to 40%, while moderate heterogeneity with *I* ^2^ of 30 to 60%, substantial heterogeneity with *I* ^2^ of 50 to 90% and considerable heterogeneity with *I* ^2^ of 75 to 100%.). Publication bias was assessed by funnel plot and Egger’s test (Egger’s test was done only if the studies number was no less than 10) [25], whereas trim and fill [26] was performed if publication bias existed. To combine indirect and direct evidence, network meta-analysis was performed to evaluate the treatment effect of all treatments. We also compared the results of traditional meta-analysis and network meta-analysis. Effect sizes were calculated by related risk (RR) and 95% confidence intervals (CIs). Meta-analysis was performed in R software version 3.2.0 [27] (with the package “meta” [28] for traditional meta-analysis and “pcnetmeta” [29] for Bayesian network meta-analysis).

## Results

### Searching result

**Fig. 1:**
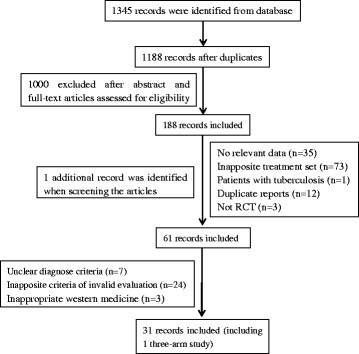
Flow chart of study selection

A total of 1345 potentially relevant citations were identified, 47 of which were degree research thesis. 157 duplicate papers were removed firstly, and 1000 papers were excluded after scanning their titles and abstracts. After screening the full texts of the included articles, 158 studies were excluded for the following reasons: no relevant data (*n* = 35), inapposite treatments set (*n* = 73), patients with tuberculosis (*n* = 1), duplicate reports (*n* = 12), not RCT (*n* = 3), unclear diagnose criteria (*n* = 7), inapposite criteria of invalid evaluation (*n* = 24), inappropriate western medicine (*n* = 3). However, one additional article [30] was identified during screening. Finally, 31 studies [17, 18, 30–58] (including1 three-arm study [18]) were included for further analysis (Fig. 1).

### Studies description

We included 31studies (Additional file 1: Table S1), all of these were conducted in China and published in Chinese. The sample size of these studies ranged from 30 to 200, and the total number of patients was 2255. The age of patients ranged from 18 to 78, and the duration of treatment was from 10 to 90 days. All studies adopted the diagnosis criteria of American CDC (1988 or 1994 edition). As acupuncture and moxibustion had many forms, such as auricular acupuncture, electroacupuncture, which were acupuncture, we treated them all as acupuncturein the present study. Therefore, the control treatments were Chinese herbal medicine, western medicine, sham-acupuncture and SAM (single acupuncture or single moxibustion).

All the included studies were divided into five groups based on the intervention in experiment and control groups: (1) CbAM (combined acupuncture and moxibustion, which meant two or more types of acupuncture and moxibustion were adopted) versus Chinese herbal medicine (*n* = 8); (2) SAM versus Chinese herbal medicine (*n* = 12); (3) SAM versus western medicine (*n* = 4); (4) SAM versus sham-acupuncture (or placebo treatment) (*n* = 4); (5) CbAM versus SAM (*n* = 5).

### Quality of the included studies

**Fig. 2:**
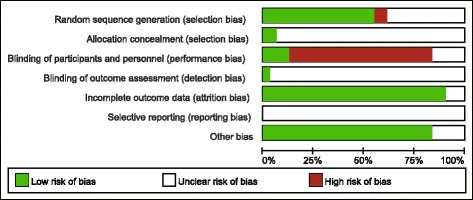
The Cochrane risk of bias

After assessing the quality of studies based on the basis of Cochrane risk of bias, we revealed that 17 trials reported random sequence generation [17, 18, 31, 33–36, 38, 39, 42, 44, 45, 49, 51, 53, 54, 58], 2 studies provided information on allocation concealment [17, 51], 4 trials described blinding of participants (single or double) [34, 39, 46, 49],1 trial described blinding of outcome assessment [39],3 trials had unclear bias of complete data [17, 33, 58] and 5 trials had unclear other biases (defined as baseline data comparability) [34, 39, 45, 52, 55]. Selective reporting bias was unclear (Fig. 2).

### Synthesis of results

#### CbAM versus Chinese herbal medicine

**Fig. 3:**
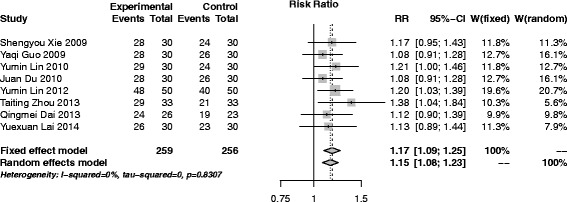
Forest plot of CbAM versus Chinese herbal medicine

Eight trials [18, 35, 36, 40, 42, 50, 52, 56], 515 participants (259 in experiment group and 256 in control) were included in this group. The pooled results of the 8 trials that compared CbAM with Chinese herbal medicine were of RR (95% CI), 1.17 (1.09-1.25), indicating that CbAM was more effective than Chinese herbal medicine (Fig. 3). There were no heterogeneity (*I* ^2^ = 0%, *p* = 0.83).

#### SAM versus Chinese herbal medicine

**Fig. 4:**
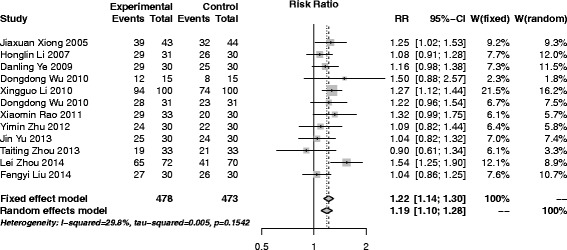
Forest plot of SAM versus Chinese herbal medicine

Twelve trials [17, 18, 31, 33, 38, 41, 44, 45, 48, 51, 54, 58], 951 participants (478 in experiment group and 473 in control) were included in this group. With only not important heterogeneity existed (*I* ^2^ = 29.8%, *p* = 0.15), the pooled result of the 8 trials was performed with fixed effect model and the result (RR (95% CI), 1.22 (1.14–1.30) showed that SAM had better effects than Chinese herbal medicine (Fig. 4). There was no publication bias after egger’s test (*p* = 0.82).

#### SAM versus western medicine

**Fig. 5:**
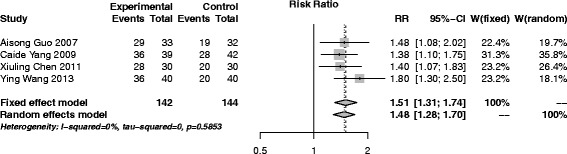
Forest plot of SAM versus western medicine

A total of 4 trials [32, 37, 47, 53] (286 participants, 142 in experiment group and 144 in control) were adopted in this group. The pooled results indicated that SAM had a better effect than western medicine (RR (95% CI), 1.51 (1.31–1.74)), with no heterogeneity among the studies (*I* ^*2*^ = 0%, *p* = 0.59) (Fig. 5).

#### SAM versus placebo treatment (sham-acupuncture)

**Fig. 6:**
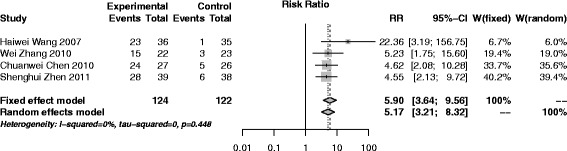
Forest plot of SAM versus placebo treatment (sham-acupuncture)

Four trials [34, 39, 46, 49] (246 participants, 124 in experiment group and 122 in control) were conducted to compare SAM with sham-acupuncture. The pooled result indicated that SAM was much more effective than sham-acupuncture (RR (95% CI), 5.90 (3.64–9.56)), with no heterogeneity (*I* ^2^ = 0%, *p* = 0.45) (Fig. 6).

#### CbAM versus SAM

**Fig. 7:**
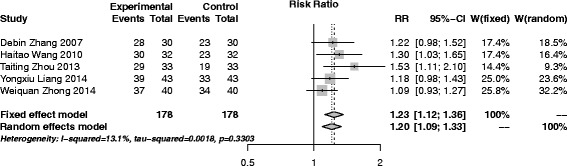
Forest plot of CbAM versus SAM

A total of 5 trials [18, 30, 43, 56, 57], 356 participants (178 in experiment group and 178 in control) were involved in this group. The pooled results revealed that CbAM was more effective than SAM (RR (95% CI), 1.23 (1.12–1.36)) (Fig. 7). The statistic *I* ^*2*^ for homogeneity of RR showed there was no heterogeneity among the studies (*I* ^*2*^ = 13.1%, *p* = 0.33).

Sensitivity analyses were performed in all traditional meta-analysis. No small sample effect existed after removing any single trial. Random effect model was also adopted and the results were similar to the fixed random effect, showing stable results (Figs. 3, 4, 5, 6 and 7).

#### Network meta-analysis

**Fig. 8:**
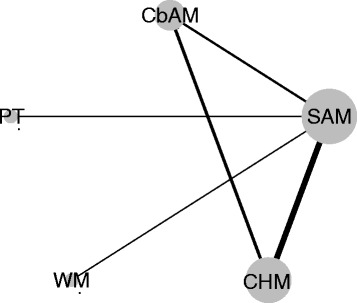
Network of involved treatments. CbAM, combined acupuncture and moxibustion; SAM, single acupuncture or single moxibustion; CHM, Chinese herbal medicine; WM, western medicine; PT, placebo treatment (sham-acupuncture)

**Fig. 9:**
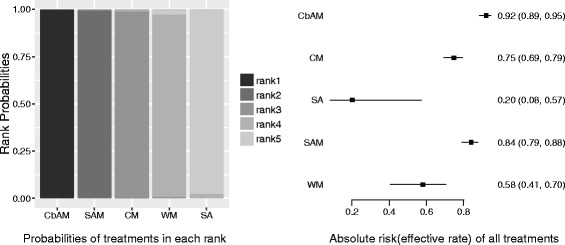
Comprehensive comparison of all treatments. *Left*, probabilities of all treatments in each rank; *right*, absolute risk (effective rate) of all treatments

The results of network meta-analysis were similar with the traditional meta-analysis (Table 1), showed that all treatments were more effective than sham-acupuncture, and the comprehensive rank (from high to low effective rate) was CbAM, SAM, Chinese herbal medicine, western medicine and sham-acupuncture (Figs. 8 and 9)

**Table 1:** Comparison of network meta-analysis and traditional meta-analysis

	CbAM		SAM		Chinese medicine		Western medicine		Placebo (Sham-acupuncture)	
Treatment	T-meta^a^	N-meta^b^	T-meta^a^	N-meta^b^	T-meta^a^	N-meta^b^	T-meta^a^	N-meta^b^	T-meta^a^	N-meta^b^
CbAM	–	–	1.23 (1.12–1.36)	1.10 (1.04–1.17)	1.17 (1.09–1.25)	1.23 (1.15–1.32)	–	1.68 (1.29–1.69)	–	5.84 (1.19–12.21)
SAM	–	0.91 (0.85–0.96)	–	–	1.22 (1.14–1.30)	1.12 (1.03–1.21)	1.51 (1.31–1.74)	1.53 (1.17–2.43)	5.90 (3.64–9.56)	5.31 (1.74–11.07)
Chinese medicine	–	0.81 (0.77–0.87)	–	0.89 (0.83–0.97)	–	–	–	1.20 (1.10–1.50)	–	4.00 (2.50–6.90)
Western medicine	–	0.62 (0.37–0.77)	–	0.68 (0.41–0.85)	–	0.76 (0.46–0.96)	–	–	–	3.20 (1.90–5.50)
placebo (sham-acupuncture)	–	0.21 (0.08–0.53)	–	0.24 (0.09–0.58)	–	0.26 (0.10–0.65)	–	0.37 (0.12–0.99)	–	–

^a^stands for traditional meta-analysis

^b^stands for network meta-analysis

#### Adverse events

Of the conducted trials, adverse events happened in four studies [17, 31, 33, 42]. In total, there was fainting during acupuncture (6 cases), feeling of acid bilges on the back (6 cases),subcutaneous hematoma (1 case), burn during moxibustion (1 case). No serious adverse events happened.

#### Acupuncture point

The acupoints were chosen with two ways, constant point and constant point plus points based on the basis of symptoms. After analysis of points adopted in trial, we found *Walking Three Miles (ST36), Spleen Locus (BL20),* and *Liver Locus (BL18)* were the three points most commonly used (Additional file 2: Figure S1).

## Discussion

In our meta-analysis, we included 31 trials for traditional and network meta-analysis. In traditional meta-analysis, CbAM and SAM were more effective than Chinese herbal medicine, western medicine and sham-acupuncture. In network meta-analysis, similar results were found. We also analysed all treatments by network meta-analysis, of which CbAM was the most effective, followed by SAM, Chinese herbal medicine, western medicine, and then sham-acupuncture.

The comparison of network meta-analysis and traditional meta-analysis depicted the same qualitative results (Table 1). It is reported that network meta-analysis can get narrower CI for which it can combine direct and indirect results [59]. In this research, similar conclusions were observed in comparison of CbAM versus SAM. However, in the comparison of SAM versus western medicine, SAM versus sham-acupuncture, the 95% CI became broader in network meta-analysis. This may be due to the inconsistency between direct and indirect comparison. The inconsistency test also showed that inconsistency existed in triangular loop of CbAM, SAM, Chinese herbal medicine, suggesting that the quantitative results of comparison needed further research although the same qualitative results were obtained.

Even though more and more evidences show the efficiency of acupuncture and moxibustion, there is still doubt that the functions of acupuncture and moxibustion are owing to placebo effect [60, 61]. In our investigation we confirmed the efficiency of acupuncture and moxibustion by traditional and network meta-analysis, showing acupuncture and moxibustion had better effects than the placebo and also Chinese herbal medicine, western medicine, and similar results of acupuncture and moxibustion were also obtained in treating chronic pain when compared with placebo treatment [62]. Although the mechanism of acupuncture and moxibustion is still unclear, and some evidences of eradicating diseases and keeping healthy by means of acupuncture and moxibustion from western medicine have been observed. In the trials of *Zhen* [63], *Xiong* [64], they found that immunity factors IL-6, IFN-γ, TNF-α changed significantly after the treatment with acupuncture and moxibustion, indicating that the treatment could strengthen the immune system. In animal experiments [65, 66], compared with normal rats, the CFS rats had lower immunity factors of IgA, IgM, IgG and IFN-γ. After the treatment of acupuncture and moxibustion, the immunity factors recovered again indicating CFS might be caused by immune function disorder, and regulating immunity may be one of the methods that acupuncture and moxibustion adopt to treat CFS.

In this research, we focused on quantitative assessment of acupuncture and moxibustion by traditional and network meta-analysis, and compared it with Chinese herbal medicine and western medicine. Even though some similar researches have been performed before [19–21], some new informations were provided, for example, network meta-analysis. We also analyzed the acupoints adopted in trials, which was found to be a little different with the previous research [19]. The head five acupoints were *Walking Three Miles (ST36)*, *Crossroad of Three Yins (SP6)*, *Hundred Meetings (GV20)*, *Back Area of Governing Vessel Meridian*, *Back Area of Urinary Bladder Meridian* [19] and *Walking Three Miles (ST36)*, *Spleen Locus (BL20)*, *Liver Locus (BL18)*, *Kidney Locus (BL23)*, *Crossroad of Three Yins (SP6)* in our research. The reasons of the difference may be the criteria of inclusions and exclusions, and that the research time was different, we enrolled more trials than the earlier research. The acupoints information could provide some suggestions when chronic fatigue syndrome patients were treated by acupuncture and moxibustion.

From our results, the traditional Chinese medicine, as a type of complementary and alternative medicine, has good effects in relief of CFS symptoms. However, the others complementary and alternative medicines need further exploration. Furthermore, a group-based self-management program for chronic fatigue syndrome patients seems to be useful in relief of fatigue severity, despite unsustained at the one-year follow-up, [67] therefore, it is needed to support CFS/ME patients to access reliable, evidence-based information outside primary care, and an online resource for patients to support self-management may be a good choice [68]. In addition, activity pacing self-management was also found to be feasible and effective in desired daily life activities and reducing fatigue in women with CFS [69].

In this meta-analysis, we concentrated on the effect of acupuncture and moxibustion, and did not include trials for Chinese herbal medicine versus western medicine. Through network meta-analysis, we found Chinese herbal medicine had better effect than western medicine, which was similar to a traditional meta-analysis of Chinese herbal medicine to western medicine [70], and these two similar results provided extra evidence that our results were relatively reliable.

## Limitations

In this review, 31 RCTs for treating CFs were identified, but the quality wasn’t high. Some factors that could influence the results were discussed below. First, the quality of methodology of the studies was poor. Only two studies provided information on allocation concealment [17, 51], four trials described blinding of participants (single or double) [34, 39, 46, 49], one trial described blinding of outcome assessment [39], and three trials had unclear bias of complete data [17, 33, 58]. These characteristics may lead to bias in selection, performance, and detection and may result in false-positive findings. However, this is a more comprehensive result so far. Second, as the limitation of sample size in some groups, such as SAM versus western medicine, some results still need further confirmations. Third, the qualitative results of some comparisons between traditional and network meta-analysis were a little different, indicating that the exact effects need further exploring. Fourth, there was potential publication bias among the included studies. All these trials were conducted in China and published in Chinese. Therefore positive results may be easier to be published. However, the heterogeneity might not be important as the *I* ^2^ in all groups which was below 30%, that showed good effects of acupuncture and moxibustion. Fifth, although the outcome measure of effective rate was widely used in many related studies. The outcome measure was really a limitation due to the reason that effective rate was not a validated outcome and the definition could be subjective. This further limits the reliability of the findings.

## Conclusions

In this systematic review, we evaluated the treatment effect of acupuncture and moxibustion comprehensively which were found to be more effective than Chinese herbal medicine, western medicine and placebo treatment (sham-acupuncture) in relieving symptoms. However, because of low quality evidence and heterogeneity, further studies are required to confirm this hypothesis.

## Additional files

Additional file 1: Table S1. The characteristics of included studies. (PDF 630 kb)

Additional file 2: Figure S1. The highest frequency acupoints adopted in studies. (PDF 123 kb)

## Abbreviations

AM: Acupuncture and moxibustion
CFS: Chronic fatigue syndrome
TCM: Traditional Chinese medicine
