# Supplementary material for: Acupuncture and moxibustion for chronic fatigue syndrome in traditional Chinese medicine: a systematic review and meta-analysis
Source: BMC Complement Altern Med. 2017 Mar 23;17:163. doi: 10.1186/s12906-017-1647-x (PMC5363012; doi:10.1186/s12906-017-1647-x)
Supplement: Supplementary file 1 — The characteristics of included studies. (PDF 630 kb) [file 12906_2017_1647_MOESM1_ESM.pdf]

Table S1. The characteristics of included studies

No.	Study(first suthor)	Year	Sample size	age	Intervention group	Control group (the third group for three-arm trial)	Treatment duration (days)	Adverse events
1	Jiaxuan Xiong	2005	87(43/44)	18-60	electroacupuncture	*liuweidihuangwan*	20	5
2	Aisong Guo	2007	65(33/32)	21-49	moxibustion	fluoxertine	30	
3	Debin Zhang	2007	60(30/30)	21-59	acupuncture,moxibustion	acupuncture	30	
4	Haiwei Wang	2007	71(36/35)	NA	acupuncture	placebo treatment	10	
5	Honglin Li	2007	61(31/30)	33-48	acupuncture	*cannanxingkoufuye*	30	5
6	Caide Yang	2009	81(39/42)	21-50	embedding	doxepin	45	
7	Danling Ye	2009	60(30/30)	20~-50	acupuncture	*xiaoyaosan*	21	
8	Shengyou Xie	2009	60(30/30)	18-50	electroacupuncture,auricular-plaster	zaorentang,ganmaidazaotang	28	
9	Yaqi Guo	2009	60(30/30)	18-60	acupuncture,auricular-plaster	*guipitang*	28	
10	Chuanwei Chen	2010	53(27/26)	21-58	acupuncture	placebo treatment	14	
11	Dongdong Wu	2010	30(15/15)	25-55	acupuncture	*guipitang*	14	
12	Dongdong Wu	2010	62(31/31)	19-54	acupuncture	*cannanxingkoufuye*	28	
13	Haitao Wang	2010	64(32/32)	21-60	acupuncture,moxibustion	acupuncture	30	
14	Juan Du	2010	60(30/30)	20-55	acupuncture,moxibustion	*guipiwan*	30	
15	Wei Zhang	2010	45(22/23)	29-78	acupuncture	placebo treatment	28	
16	Xingguo Li	2010	200(100/100)	22-56	acupuncture	*shiquandabutang*	28	
17	Yumin Lin	2010	60(30/30)	30-46	acupuncture,moxibustion	*guipiwan*	21	3
18	Shenghui Zhen	2011	77(39/38)	35-43	acupuncture	placebo treatment	28	
19	Xiaomin Rao	2011	63(33/30)	24-36	moxibustion	chinese medicine	30	
20	Xiuling Chen	2011	60(30/30)	31-39	moxibustion	vitamin	28	
21	Yimin Zhu	2012	60(30/30)	19-48	abdominal acupuncture	*xiaoyaosan*	17	
22	Yumin Lin	2012	100(50/50)	NA	acupuncture,moxibustion	*guipiwan*	21	
23	Jin Yu	2013	60(30/30)	20-~50	acupuncture	*xiaoyaosan*	21	
24	Qingmei Dai	2013	49(26/23)	30-55	acupuncture,moxibustion	*xiaoyaowan*	42	
25	Taiting Zhou	2013	99(33/33/33)	27-53	acupuncture,moxibustion	*guipitang*(electroacupuncture)	36	
26	Ying Wang	2013	80(40/40)	20-57	moxibustion	fluoxertine hydrochloride	20	
27	Fengyi Liu	2014	60(30/30)	26-40	embedding	*xiaoyaowan*	90	1
28	Lei Zhou	2014	142(72/70)	18-56	embedding	*qijudihuangwan*	90	
29	Weiquan Zhong	2014	80(40/40)	22-40	acupuncture,moxibustion	electroacupuncture	40	

30	Yongxiu Liang	2014	86(43/43)	27-59	embedding, moxibustion	acupuncture	35	
31	Yuexuan Lai	2014	60(30/30)	26-38	acupuncture,moxibustion	*xiaoyaowan*	28	
