# Supplementary figures and images for: Acupuncture and moxibustion for chronic fatigue syndrome in traditional Chinese medicine: a systematic review and meta-analysis
Source: BMC Complement Altern Med. 2017 Mar 23;17:163. doi: 10.1186/s12906-017-1647-x (PMC5363012; doi:10.1186/s12906-017-1647-x)

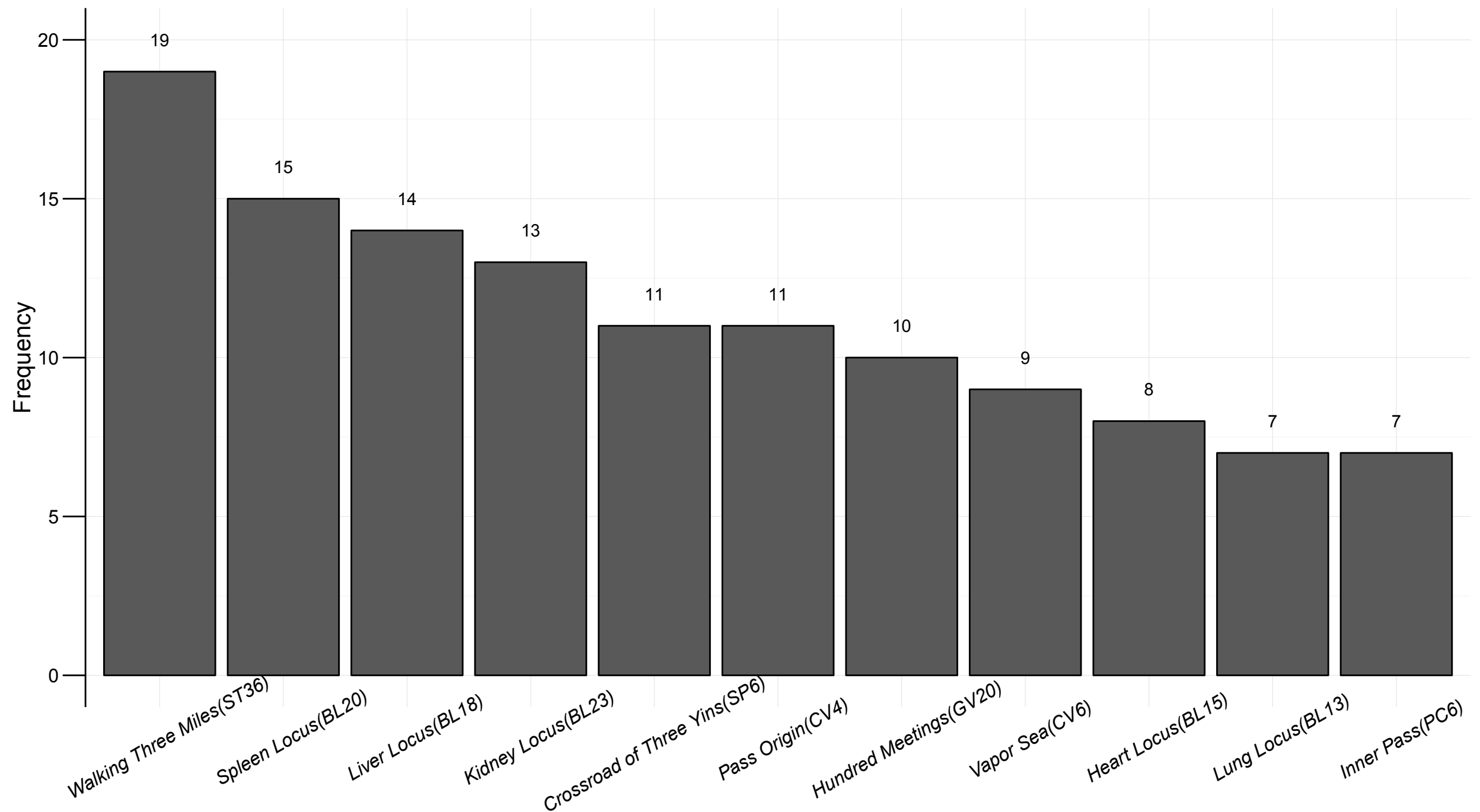

Fig.S1 The highest frequency acupoints adopted in studies

Supplement: Supplementary file 2 — The highest frequency acupoints adopted in studies. (PDF 123 kb) [file 12906_2017_1647_MOESM2_ESM.pdf]
